# Valorization of local agricultural by-products as nutritional substrates for *Tenebrio molitor* larvae: A sustainable approach to alternative protein production

**DOI:** 10.1007/s11356-024-33564-8

**Published:** 2024-05-14

**Authors:** Mariastela Vrontaki, Christina Adamaki-Sotiraki, Christos I. Rumbos, Anastasios Anastasiadis, Christos G. Athanassiou

**Affiliations:** 1 Laboratory of Entomology and Agricultural Zoology, Department of Agriculture, Crop Production and Rural Environment, University of Thessaly, Phytokou Str, 38446 Volos, Greece; 2 Animal Feed Anastasiadi Single Member P.C, 61100 Akropotamia Kilkis, Greece

**Keywords:** Agricultural by-products, Valorization, Feeding substrates, Insect farming, *Tenebrio molitor*, Sustainable protein source, Circular economy

## Abstract

In pursuit of sustainable protein sources, the agricultural sector and emerging edible insect industry intersect in the valorization of agricultural by-products. Establishing a mutually beneficial relationship involves utilizing agricultural by-products as feeding substrates for insect farming, potentially enhancing the sustainability of both sectors. In the present study, by-products from beer, rice, oat, maize, sunflower, and lucerne, as well as mill residues and spent mushroom substrate from the regions of Thessaly and Central Macedonia (Greece) were investigated as nutritional sources for the larvae of the yellow mealworm (*Tenebrio molitor*). Results show that the suitability of the tested by-products for rearing *T. molitor* larvae varies greatly, with larvae surviving better in some by-products than in others. The highest survival rate and the highest weight of larvae were recorded for larvae reared on rice bran, spent grains, and oat by-products. Similarly, high feed conversion and growth rate were observed when the larvae were fed with rice bran and spent grains. Thus, this research promotes cost-effective and sustainable *T. molitor* rearing, aligning with circular economy principles.

## Introduction

The aquaculture and the livestock industry are currently making systematic efforts to evaluate novel, alternative, and sustainable protein sources that could partially replace the traditional protein sources (e.g., fish meal and fish oil, soymeal) which are linked with certain environmental constraints (e.g. deforestation, overfishing, etc.) (Tacon and Metian 2008; Barona et al. 2010; Tilman and Clark 2014; Henchion et al. 2017; Gorissen and Witard 2018; Kim et al. 2019). In addition, the human population is constantly growing with projections indicating that by 2050, approximately 10 billion people will need to be fed (Nations 2019), while it has also been calculated that by 2050, the food sector will have to increase its production by 70% compared to 2009, in order to meet the nutritional needs of the human population (FAO 2009). The increased demand for grains and legumes as food and feed ingredients results in subsequent rise in prices (Fan and Pandya-Lorch 2012). Therefore, the scientific community is in searching for innovative, sustainable, and affordable sources of protein. Although several novel protein sources have been evaluated in this direction (Salter and Lopez-Viso 2021), insects represent one of the most promising sources of high-quality protein (Koutsos et al. 2019; Patel et al. 2019).

Insects have important advantages for both human and livestock nutrition due to their high protein content, amino acids, lipids and various micronutrients (Defoliart 1995; Rumpold and Schlüter 2013; Makkar 2018). Moreover, their low requirements for land and water, as well as their low greenhouse gas emissions and their high feed conversion ratio, render insect rearing a highly environmental-friendly process, compared with conventional livestock production (Van Huis et al. 2013; Jansson and Berggren 2015;). Along the same lines, due to their ability to biodegrade waste generated in the food industry, insect production is fully aligned with circular economy strategies that are currently promoted by EU (Kelemu et al. 2015; Sangiorgio et al. 2022).

Several insect species have been already successfully utilized as waste management agents for materials of both plant and animal origin (Ramos-Elorduy et al. 2002; Harsányi et al. 2020). Agricultural production results in the generation of a wide range of by-products such as broken kernels and seeds, husk, and shells, that are often discarded, or used as low-economic value feedstocks (Galanakis 2013; Pan et al. 2019). In this context, the inclusion of agricultural by-products in insect diets can significantly reduce the insect production cost, since the feed cost substantially contributes to the total production (Roffeis et al. 2018; Varelas 2019; Gasco et al. 2020). This approach has been already widely evaluated for several insect species (i.e. *Alphitobius diaperinus* (Panzer) (Coleoptera: Tenebrionidae), *Tenebrio molitor* (L.) (Coleoptera: Tenebrionidae), *Hermetia illucens* (L.) (Diptera: Stratiomyidae)) that are massively produced for food and feed, particularly exploiting grains and related amylaceous commodities (Ruschioni et al. 2020; Scala et al. 2020; Gourgouta et al. 2022; Rumbos et al. 2022).

The yellow mealworm, *Tenebrio molitor* L. (Coleoptera: Tenebrionidae), has been extensively studied for its potential to be utilized as a nutrient source for farmed fish (Iaconisi et al. 2017; Jeong et al. 2020), poultry (Khan et al. 2018; Mastoraki et al. 2020; Vasilopoulos et al. 2023), pigs (Jin et al. 2016; Zacharis et al. 2023) and other livestock animals. In 2017, *T. molitor* was listed among the approved insect species as aquafeed ingredients (EC 2017). Moreover, in 2021, *T. molitor* larvae were authorized to be used as ingredients of poultry and swine feeds (EC 2021), as well as for human consumption (EFSA 2021). Several studies so far have evaluated the development of *T. molitor* larvae fed various by-product-based diets with promising results (Mancini et al. 2019; Oonincx et al. 2019; Stull et al. 2019; Rumbos et al. 2022). In this framework, the objective of the present study aims to evaluate nine agricultural organic side-streams, from the production of beer, rice, oat, barley, lucerne and maize, which are massively produced in the regions of Thessaly and Central Macedonia, Greece, as feeding substrates of *T. molitor* larvae.

## Materials & methods

### Insects

Insect stock colonies were maintained in plastic insect breeding trays (60 × 40 × 14.5 cm) (Beekenkamp Verpakkingen BV, Maasdijk, The Netherlands) in the pilot-scale insect rearing unit of the Laboratory of Entomology and Agricultural Zoology (LEAZ) of University of Thessaly, Greece, under constant conditions, i.e., 27 ± 0.5 °C, 60 ± 5% relative humidity (RH) and continuous darkness. A thorough description of the Greek strain utilized for experimentation is provided by Rumbos et al. (2021). Wheat bran was used as feedstock of *T. molitor* larvae was, while agar (20 g/L) was provided as a moisture source to both adults and larvae three times per week.

Ten days old larvae were used for the experiments. To obtain newly hatched larvae, adults were allowed to oviposit on white wheat flour for a few days. After this time, all adults were removed, the eggs were collected by sieving and newly hatched larvae were allowed to live on white wheat flour for 10 days until the start of the experiments. To separate larvae from the flour and to select larvae of similar size for the experiments, the flour containing the larvae was sieved by hand through 850 and 600 μm sieves. Larvae that passed through the 850 μm sieve and were retained by the 600 μm sieve were used for the bioassay. In this way, 6th- and 7th-instar larvae were selected for experimentation, according to their head capsule size (Morales-Ramos et al. 2015) Fig 1.

**Fig. 1:**
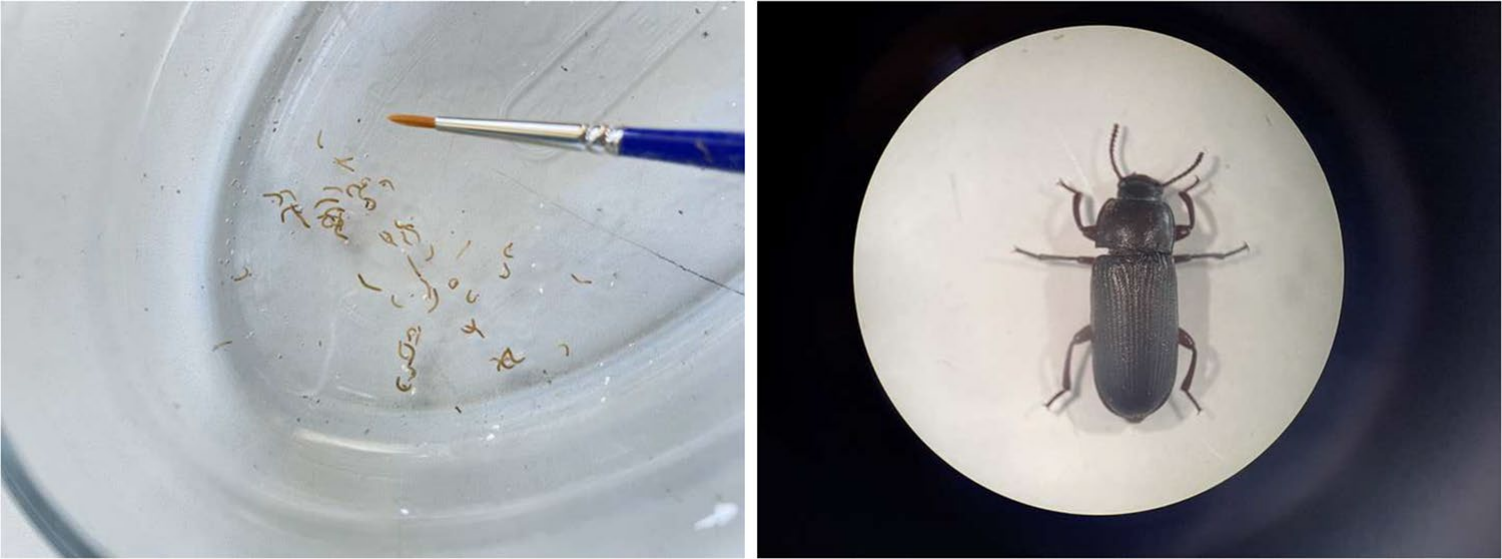
Left: Newly hatched larvae of *Tenebrio molitor*. Right: Adult stage of *Tenebrio molitor*

### By-products and experimental set up

By-products originating from the production of beer, rice, oat, barley, lucerne and maize were evaluated (Table 1). The following by-products were tested in detail: sunflower by-product (small and broken seeds)*,* lucerne by-product (in pellets), oat by-product (beard hairs, husks, small oat seeds), spent grains (in pellets), maize by-product (remains of the seed cleaning process), rice bran, rice husk (the hard protecting coverings of rice grains that are removed from the rice kernels during rice production), spent mushroom substrate (the remaining substrate after the harvest of mushrooms) and the residues of the animal feed grinding process (75% maize, 15% barley, 5% soft wheat, 5% other grains)*.* All by-products were grounded using thermomix (TM31-96 1C, Vorwerk Elektrowerke GmbH & Co. K, Wuppertal, Germany), except for rice bran and the animal feed mill residues, which were already in powder form.

**Table 1:** Dry matter (%), nitrogen (% DM) and ash (% DM) content of nine agricultural by-products and wheat bran (control)

By-products	Dry matter (%)	Nitrogen (%)	Ash (%)
Wheat bran (control)	85.9	3.0	4.7
Sunflower by-product	96.7	3.4	3.8
Lucerne by-product	94.8	2.5	4.2
Oat by-product	97.1	2.1	3.8
Spent grains	95.6	3.4	5.1
Maize by-product	95.8	1.3	4.9
Mill residues	93.3	0.8	4.2
Rice bran	91.7	2.6	7.7
Rice husk	96.1	1.0	4.8
Spent mushroom substrate	97.9	1.1	13.0

Within each column, means followed by the same lowercase letter are not significantly different

All by-products were screened individually in cylindrical, plastic vials (7.5 cm in diameter, 8.8 cm in height) as an experimental unit, whereas wheat bran served as control. A group of 50 ten days old larvae was placed into each vial along with four grams of each by-product. Previously, larvae were weighted as a group to estimate the initial individual larval weight at the beginning of the trial. Larvae were provided with agar cubes (1 × 1 × 1 cm) three times per week, as noted above, and were allowed to feed undisturbed for 2 weeks after the bioassay began. After this period, the larvae were separated from the substrate and their survival and weight as a group was recorded. Then, they were returned to the substrate and larval survival and growth was determined bi-weekly until the emergence of the first pupa. All vials were visually inspected three times a week for lack of food. If the feed got depleted, more feed was added and the feed amount added was recorded. There were six replicates for each treatment. The experimental conditions were the same as the breeding conditions, i.e., 27 ± 0.5 °C, 60 ± 5% relative humidity (RH) and continuous darkness.

### Proximate composition

The dry matter of the by-products was determined by thermal drying in an oven at 105 °C for 24 h until constant weight was reached. The crude protein content was determined by Kjeldahl analyses (Behr Labor-Technik GmbH, Germany), while the ash content was determined by dry ashing in porcelain crucibles in a muffle furnace (Nabertherm L9/12/ C6, Lilienthal, Germany) at 600 °C for 5 h (Karapanagiotidis et al. 2019).

### Calculations

The individual larval weight, survival rate and development time were determined for each vial. Individual larval weight (ILW) was calculated by dividing the total weight of the live larvae with their number. Survival rate was calculated by dividing the number of the live larvae with the original number of the larvae and multiplying it by 100%. The larval development time was calculated as the number of days between the start of the experiment (when each group of 50 larvae was inserted in each vial), and the day that each vial was harvested (when the first pupa appeared in each vial).

The Feed Conversion Ratio (FCR) was calculated using the following equation:

FCR = Feed consumed / Larval weight gained.

and corresponds to the amount of feed needed to obtain one Kg of larval biomass. In addition, the Efficiency of Conversion of Ingested food (ECI) was calculated as:

ECI = (Larval weight gained / Feed consumed) × 100%,

While the Specific Growth Rate (SGR) was given by the equation:

SGR = 100 × (lnFBW – lnIBW) / days,

where FBW and IBW stand for final and initial body weight, respectively. FCR and SGR were calculated on a wet weight basis, whereas ECI on a dry matter basis (Waldbauer 1968). Agar weight was excluded from the calculations.

### Statistical analysis

Statistical analysis for final individual larval weight, final survival rate, development time, FCR, ECI and SGR was done with SPSS 26.0 (IBM Corporation, Armonk, NY, USA). The data were first tested for normality and homogeneity of variances using Levene’s tests and Shapiro–Wilk tests, respectively. Since data were not normally distributed, non-parametric analyses were performed. In order to determine whether there were significant differences (*P* < 0.05) among treatments, the Kruskal–Wallis H test was performed, followed by Dunn multiple comparisons for post-hoc testing.

## Results

### Proximate composition of the by-products

All by-products had a high dry matter content (85.9–97.9%), while the nitrogen and ash content varied depending on the by-product (0.8–3.4% and 3.9–13.3%, respectively) (Table 1). Most of the by-products had a high nitrogen content (> 2.1%), except for mill residues (0.8%), rice husk (1.0%), spent mushroom substrate (1.1%) and maize by-product (1.3%). The ash content refers to the minerals contained in the by-products, with the highest percentage of ash content found in the spent mushroom substrate (13%).

### Survival rate

**Fig. 2:**
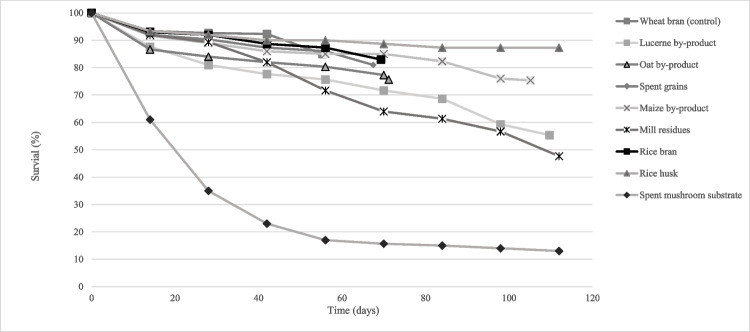
Survival rate (%) after 2, 4, 6, 8, 10, 12, 14 and 16 weeks of development of *Tenebrio molitor* larvae reared with nine agricultural by-products and wheat bran (control). For all treatments *n* = 6, except for the sunflower by-product, for which *n* = 1 after week 6, due to increased larval mortality

In all feed treatments, the survival rate gradually decreased over the course of the experiment. Statistically significant differences between treatments were found in the final survival rate (df = 8; *P* < 0.001), which ranged from 0.7% (sunflower by-product) to 85.0% (spent grains) (Fig. 2; Table 2). The results of the experiment revealed that larvae on rice bran, maize, oat by-product and wheat bran (control) had the highest survival rate, which was more than 79.7%. However, the larvae fed on the spent mushroom substrate had a very low survival rate of only 13.0%. Likewise the larvae fed on the sunflower by-product had the lowest survival rate of only 0.7%. By week 6, all the larvae that were in vials containing the sunflower by-product had died, except for one vial replicate.

**Table 2:** Survival rate (% ± SE), final individual larval weight (FILW, mg ± SE), development time (DT, days ± SE) of *Tenebrio molitor* larvae reared with nine agricultural by-products and wheat bran (control), at the end of the bioassay. For all treatments *n* = 6, except for the sunflower by-product, for which *n* = 1 after week 6, due to increased larval mortality

By-products	Survival (%)	FILW (mg)	DT (days)
Wheat bran (control)	92.0 ± 4.9 a	107.8 ± 10 ab	55.3 ± 1.6 a
Sunflower by-product	0.7	109.8	110.0
Lucerne by-product	55.3 ± 2.3 bc	73.5 ± 7.2 bc	109.7 ± 3.5 ab
Oat by-product	79.7 ± 10.8 ab	102.9 ± 8.2 ab	71.7 ± 3.4 a
Spent grains	85.0 ± 7.5 ab	107.0 ± 12.4 ab	67.5 ± 6.2 a
Maize by-product	82.7 ± 6.4 ab	88.5 ± 6.7 bc	105.2 ± 7.2 ab
Mill residues	47.7 ± 11.0 c	18.8 ± 2.9 c	126.0 ± 0.0 b
Rice bran	83.0 ± 6.0 a	120.2 ± 12.3 a	69.3 ± 3.1 a
Rice husk	43.7 ± 2.4 c	30.2 ± 2.5 c	126.0 ± 0.0 b
Spent mushroom substrate	13.0 ± 7.9 c	6.9 ± 2.1 e	126.0 ± 0.0 b

Within each column, means followed by the same lowercase letter are not significantly different

### Development time

The development time varied between the larvae reared with the tested by-products and wheat bran (control) and ranged from 55 to 126 days (df = 8; *P* < 0.001). The shortest development time (55 days) was observed with wheat bran (control) followed by rice bran (69 days), spent grains (67 days) and oat by-product (71 days), although the latter three did not differ significantly from the control. When the larvae were fed on mill residues, rice husk and spent mushroom substrate, they grew more slowly (up to 120 days) (Table 2).

### Individual larval weight

**Fig. 3:**
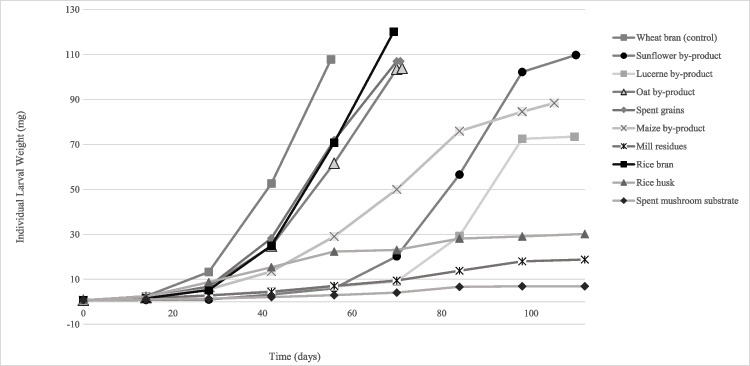
Individual larval weight (mg) after 2, 4, 6, 8, 10, 12, 14 and 16 weeks of development of *Tenebrio molitor* larvae reared with nine agricultural by -products and wheat bran (control). For all treatments *n* = 6, except for the sunflower by-product, for which *n* = 1 after week 6, due to increased larval mortality

Larval growth, expressed as individual larval weight, varied between rearing substrates (df = 8; *P* < 0.001). The final larval weight ranged between 6.9 mg (spent mushroom substrate) and 120 mg (rice bran) (Fig. 3; Table 2). Larvae reared on rice bran, wheat bran (control), spent grains and oat by-product reached a high final larval weight, while larvae fed on spent mushroom substrate, mill residues, lucerne by-product and rice husk, grew smaller (< 30.2 mg).

### Feed conversion ratio (FCR), efficiency of conversion of ingested food (ECI) and specific growth rate (SGR)

FCR (df = 8; *P* < 0.001) and ECI (df = 7; *P* < 0.001) varied between treatments and ranged from 2.2 (wheat bran) to 146.8 (spent mushroom substrate) and from 2.4% (mill residues) to 46.7% (wheat bran, control), respectively (Table 3). Low FCRs and high ECIs indicate that the larvae can utilise the feed efficiently. Larvae reared on wheat bran had in the lowest FCRs and the highest ECIs (2.2 and 46.7%, respectively), while similar values were found for larvae fed on rice bran (2.6 and 38.9%, respectively) and spent grains (2.8 and 36.4%, respectively). Larvae reared on spent mushroom by-product achieved an extremely high FCR (146.8), while the lowest ECI was observed in larvae fed with mill residues (2.4%), indicating that these diets were not efficiently converted to body mass. The SGR varied between 1.7% (spent mushroom substrate) and 9.4% (wheat bran) (df = 8; *P* < 0.001) (Table 3). Ηigh SGR values, similar to the control, were calculated for larvae reared on spent grains (7.3%) and rice bran (7.5%), while rearing larvae on lucerne by-product, mill residues, rice husk and spent mushroom substrate resulted in low SGR values (< 4.3%).

**Table 3:** Feed conversion ratio (FCR), efficiency of ingested food conversion (ECI, %), specific growth rate (%/day) of *Tenebrio molitor* larvae reared with nine agricultural by-products and wheat bran (control), at the end of the experiment. For all treatments *n* = 6, except for the sunflower by-product, for which *n* = 1 after week 6, due to increased larval mortality

By-products	FCR	ECI (%)	SGR (%/day)
Wheat bran (control)	2.2 ± 0.3 a	46.7 ± 7.0 a	9.4 ± 0.2 a
Sunflower by-product	36.4	2.4	4.7
Lucerne by-product	8.0 ± 0.6 bcde	12.3 ± 1.0 def	4.2 ± 0.1 bcde
Oat by-product	3.2 ± 0.4 abcd	31.4 ± 4.4 bcd	6.9 ± 0.3 bc
Spent grains	2.8 ± 0.4 abc	36.4 ± 4.7 abc	7.5 ± 0.6 ab
Maize by-product	6.8 ± 2.6 cd	21.0 ± 1.4 cde	4.5 ± 0.5 bcd
Mill residues	39.8 ± 7.9 e	2.4 ± 0.5 f	2.6 ± 0.1 de
Rice bran	2.6 ± 0.3 ab	38.9 ± 4.5 ab	7.3 ± 0.3 ab
Rice husk	10.7 ± 0.9 de	9.1 ± 0.7 ef	2.9 ± 0 cde
Spent mushroom substrate	146.8 ± 128.9 e	not defined*	1.7 ± 0.3 e

Within each column, means followed by the same lowercase letter are not significantly different

## Discussion

This study has found that the larvae of *T. molitor* can thrive and develop well on various by-products. The results showed that rice bran, spent grains, and oat by-product sufficiently supported the larval performance in terms of survival, weight gain and development time. The larvae fed with rice bran and spent grains showed the best values for feed conversion ratio (FCR), efficiency of conversion of ingested food (ECI) and specific growth rate (SGR). It is evident from the high number of recent publications that the upcycling of side-streams and by-products as feeding substrates for edible insects has recently been a focal point of research (Harsányi et al. 2020; Morales-Ramos et al. 2020; Rumbos et al. 2020b, 2022; Bordiean et al. 2022; Kotsou et al. 2023). Furthermore, we evaluated the growth and performance of *T. molitor* larvae on a variety of by-products produced in the regions of Thessaly and Central Macedonia, Greece, with a particular focus on by-products generated during rice production (bran layer and husk). Rice is a cereal that is produced on a large scale worldwide and is the second most produced agricultural commodity after wheat. According to FAO (2023), its production has increased worldwide in recent years. Several projects are already working to promote sustainable rice production (Bodie et al. 2019). Thus, the utilization of its by-products in alternative sustainable industries, such as insect farming, could establish a win–win relationship.

Rice bran is rich in proteins, lipids, fibers, and antioxidants. The main fatty acids of rice bran are the palmitic acid, the linoleic acid, and the oleic acid (Bodie et al. 2019). Rice bran also contains minerals such as iron, calcium, and phosphorus, vitamin E and B, while it also contains two unique compounds; oryzanol and tocopherol (Manickavasagan et al. 2017; Bodie et al. 2019). This study shows that the larvae of *T. molitor* larvae show a clear preference for rice bran in terms of growth and survival. Rice husk, another by-product of the rice production, was found to have high mortality and low larval weight. Rice husk serve to protect the seed during germination, and consists of phenolic compounds, namely silica and lignin. Our results align with the findings of the study of Vachon et al. (2020) who reported that lignin-rich streams can repel insect species, such as the rice weevil, *Sitophilus oryzae* (Coleoptera: Curculionidae), and the Indian meal moth, *Plodia interpunctella* (Lepidoptera: Pyralidae). Focusing on the stored product insects, although from another perspective, before its potential as food and feed was recognized, *T. molitor* had already been classified as one of the secondary stored product pests (Hagstrum  2016). Based on that, reported data for other stored product beetles, such as *Tribolium castaneum* (Coleoptera: Tenebrionidae), illustrate that different feeding substrates affect their development and growth (Wong and Lee 2011; Đukić et al. 2016). Indicatively, Wong and Lee (2011) investigated the larval growth of *T. castaneum* on atta flour, wheat flour, self-rising flour, rice flour, custard powder, corn flour, tapioca starch, and potato starch and noted that larvae developed faster in atta flour and slower in potato starch. Similarly, Đukić et al. (2016) stated that larvae were not able to grow on protein-rich diets such as sunflower meal, soybean concentrate, and corn gluten.

Apart from the by-products of the rice production, *T. molitor* larvae grew well on oat and maize by-products, as well as on spent grains. Similarly, Rumbos et al. (2022) investigated the larval performance of *T. molitor* on a variety of agricultural by-products and reported that oat and barley by-products were the most suitable for the larvae. Among the by-products tested in our study, lucerne did not support efficiently for *T. molitor* larvae, which is in accordance with the studies of Rumbos et al. (2021) and Langston et al. (2023). In accordance with our results, Li et al. (2020) evaluated the larval performance of *T. molitor* on five different mushroom substrates and reported that the young larvae did not survive on four out of five substrates tested. Similarly, Riudavets et al. (2020) reported high larval mortality and slow development time for *T. molitor* larvae fed on feed mill products. Remarkably, the larval weight of the larvae reared on feed mill residues was similar to that of the control diet. In our study, we also observed a similar pattern for the larvae reared on sunflower by-product (i.e., high mortality and slow development) but high final larval weight. Interestingly, Rumbos et al. (2022) reported good larval performance on sunflower meal, which is in contrast with the results of our study for larvae reared on sunflower by-product. In addition, the FCR values reported by Rumbos et al. (2022) reported for oat and sunflower by-products were lower compared to those reported in the present study. These differences may be due to the fact that the composition of by-products may not always be the same; varies from batch to batch. In our study, the lowest FCR values were found for three by-products: rice bran, oat by-product and spent grains.

In a more recent study, Rumbos et al. (2021) reported that *T. molitor* larvae grew better and faster on by-products that had the highest protein content, i.e., triticale and lupin by-products. However, this is not always the case, as in our study, rice bran and oat by-product had a high nitrogen content close to that of the control, but the highest nitrogen content was recorded for the sunflower by-product and spent grains. Moreover, larval survival and growth was hampered on sunflower by-product. Those results could be explained by the fact that other factors such as fat content, amino acids, vitamins, and minerals can also have a decisive influence on larval growth (Han and Dingemanse 2017; Rumbos et al. 2020a).

Overall, the utilization of agricultural by-products as a feed source for *T. molitor* larvae offers numerous advantages that contribute to sustainability and efficiency in insect farming practices. Firstly, throughout the incorporation of locally produced agricultural by-products into insect feed, a reduction in reliance on traditional livestock farming and a mitigation of the environmental impact is achieved, while at the same time sustainability is promoted. Moreover, the integration of agricultural by-products into insect diets contributes to waste management by the inclusion of organic side-streams into the food chain. This approach proves to be cost-effective as the organic wastes would be, otherwise, discarded, while simultaneously the insect production cost is significantly reduced (Tacon and Mentian 2008). An additional positive aspect is the enhancement of *T. molitor* larval performance (Oonincx et al. 2019; Morales-Ramos et al. 2020; Montalbán et al. 2022). Importantly, the long-term usage of agricultural by-products fosters a circular economy model, where resources are continuously recycled and utilized efficiently (Ingrao et al. 2018; Gasco et al. 2020).

## Conclusions

Considering the excellent performance of *T. molitor* larvae on rice bran, spent grains, and oat by-products, it is apparent that these specific substrates can be further utilized as components of insect diets. It is possible that compound diets containing a variety of substrates could be more nutritionally balanced, meeting the nutritional requirements of insects. Therefore, in order to develop a complete diet for *T. molitor* larvae, it is suggested to combine multiple by-products as diet ingredients. Thus, both insect nutritional requirements, as well as the nutrient content of the selected by-products, should be further investigated.
